# Psychological Impacts on Patients with Alcohol Use Disorder: a Study in Southern Taiwan with Alcohol Use Disorders Identification Test (AUDIT) in 2020

**DOI:** 10.1192/j.eurpsy.2022.620

**Published:** 2022-09-01

**Authors:** C.Y. Chu, S.-C. Wang, C.-H. Lee, C.-M. Cheng

**Affiliations:** 1 Jianan Psychiatric Center, Ministry of Health and Welfare, Forensic And Addiction Psychiatry, Tainan, Taiwan; 2 Jianan Psychiatric Center, Ministry Health and Welfare, Forensic And Addiction Psychiatry, Tainan, Taiwan; 3 Jianan Psychiatric Center, Ministry Health and Welfare, Consultant Physician, Tainan, Taiwan

**Keywords:** alcohol use disorder (AUD), AUDIT, psychological impact, Multiple linear regression (MLR)

## Abstract

**Introduction:**

Alcohol consumption is a risk factor for various comorbidities, such as cirrhosis, chronic sclerosing stomatitis, and neuropsychiatric disorders.

**Objectives:**

Our study examined the associations between psychological factors and alcohol addiction of the individuals with alcohol use disorder (AUD) in Southern Taiwan.

**Methods:**

Demographic information as well as suicidal history and sources of stress were collected from 177 participants. The extent of alcoholism was assessed by AUDIT questionnaire. Demographic and linear regression analyses were performed with the Statistical Software Stata version 12.0 (StataCorp LP, College Station, TX, USA).

**Results:**

Demographic data, suicidal history and the causes of stress of patients divided by AUDIT scores are shown in **Table 1**. Among 177 participants, 17 (9.6%) had suicidal thoughts, 4 (2.3%) had suicide plan, 22 (12.5%) self-injured, and four-fifth of patients lived under pressure. Patients who self-harmed were with significant lower AUDIT scores of -7.24 (95% CI: -11.49 – -3.00) **(Table. 2)**. The AUDIT scores of patients with physical stress, interpersonal difficulties and loneliness increased significantly by 6.71 (95% CI: 3.19 – 10.30), 6.14 (95% CI: 2.15 – 10.13) and 5.02 (95% CI: 0.93 – 9.11), respectively **(Table. 3)**.

**Conclusions:**

Our findings indicated negative correlation with alcohol use and auto-inflicted injury. However, previous study showed systematic assessment of the association between suicide and AUD, and considered alcohol an important risk factor for suicide, which is related to mental health and affected by different genders and drinking patterns. Our results may provide reference for estimation of the alcohol-related psychological effects in Taiwan.

**Disclosure:**

No significant relationships.

